# Which Bundles of Features in a Web-Based Personally Controlled Health Management System Are Associated With Consumer Help-Seeking Behaviors for Physical and Emotional Well-Being?

**DOI:** 10.2196/jmir.2414

**Published:** 2013-05-06

**Authors:** Annie YS Lau, Judith Proudfoot, Annie Andrews, Siaw-Teng Liaw, Jacinta Crimmins, Amaël Arguel, Enrico Coiera

**Affiliations:** ^1^Centre for Health Informatics, Australian Institute of Health Innovation, University of New South WalesSydneyAustralia; ^2^School of Psychiatry, University of New South Wales and Black Dog InstituteSydneyAustralia; ^3^UNSW Counselling and Psychological Services, University of New South WalesSydneyAustralia; ^4^School of Public Health & Community Medicine, UNSW Centre for Primary Health Care and Equity, University of New South WalesSydneyAustralia; ^5^University Health Service, University of New South WalesSydneyAustralia

**Keywords:** personal health record, Web-based intervention, health service, help-seeking, emotional well-being, physical well-being, preventative health, eHealth, consumer, university

## Abstract

**Background:**

Personally controlled health management systems (PCHMS), which include a personal health record (PHR), health management tools, and consumer resources, represent the next stage in consumer eHealth systems. It is still unclear, however, what features contribute to an engaging and efficacious PCHMS.

**Objective:**

To identify features in a Web-based PCHMS that are associated with consumer utilization of primary care and counselling services, and help-seeking rates for physical and emotional well-being concerns.

**Methods:**

A one-group pre/posttest online prospective study was conducted on a university campus to measure use of a PCHMS for physical and emotional well-being needs during a university academic semester (July to November 2011). The PCHMS integrated an untethered personal health record (PHR) with well-being journeys, social forums, polls, diaries, and online messaging links with a health service provider, where journeys provide information for consumer participants to engage with clinicians and health services in an actionable way. 1985 students and staff aged 18 and above with access to the Internet were recruited online. Logistic regression, the Pearson product-moment correlation coefficient, and chi-square analyses were used to associate participants’ help-seeking behaviors and health service utilization with PCHMS usage among the 709 participants eligible for analysis.

**Results:**

A dose-response association was detected between the number of times a user logged into the PCHMS and the number of visits to a health care professional (*P*=.01), to the university counselling service (*P*=.03), and help-seeking rates (formal or informal) for emotional well-being matters (*P*=.03). No significant association was detected between participant pre-study characteristics or well-being ratings at different PCHMS login frequencies. Health service utilization was strongly correlated with use of a bundle of features including: online appointment booking (primary care: OR 1.74, 95% CI 1.01-3.00; counselling: OR 6.04, 95% CI 2.30-15.85), personal health record (health care professional: OR 2.82, 95% CI 1.63-4.89), the poll (health care professional: OR 1.47, 95% CI 1.02-2.12), and diary (counselling: OR 4.92, 95% CI 1.40-17.35). Help-seeking for physical well-being matters was only correlated with use of the personal health record (OR 1.73, 95% CI 1.18-2.53). Help-seeking for emotional well-being concerns (including visits to the university counselling service) was correlated with a bundle comprising the poll (formal or informal help-seeking: OR 1.03, 95% CI 1.00-1.05), diary (counselling: OR 4.92, 95% CI 1.40-17.35), and online appointment booking (counselling: OR 6.04, 95% CI 2.30-15.85).

**Conclusions:**

Frequent usage of a PCHMS was significantly associated with increased consumer health service utilization and help-seeking rates for emotional health matters in a university sample. Different bundles of PCHMS features were associated with physical and emotional well-being matters. PCHMS appears to be a promising mechanism to engage consumers in help-seeking or health service utilization for physical and emotional well-being matters.

## Introduction

Worldwide, governments have made multibillion dollar investments in eHealth to modernize health services delivery, with many questions still unanswered about the uptake, benefits, and cost-effectiveness of these investments [1,2]. In particular, personal health records (PHRs) now form a crucial component in many large-scale national eHealth reform strategies. However, uptake and utilization of PHRs is not as widespread as anticipated [1,2], and there are often gaps between proposed and actual benefits [3]. Finding approaches that effectively engage consumers in the use of PHRs, with the intention to improve health outcomes and reduce attrition rates, remains a high priority in consumer eHealth research [4-6].

PHRs have been advocated as the next generation tool that significantly improves consumers’ health behaviors and health outcomes [7]. In a key discussion on personal health records (PHRs) presented by Tang and colleagues, a PHR is an electronic application through which individuals can access, manage, and share their health information [8]. A tethered PHR allows patients to view their own health information that is stored in their health care provider’s electronic health record, whereas an untethered PHR is a stand-alone application that does not connect with any other system [8]. A personally controlled health management system (PCHMS) in this instance is a health management system that allows consumers and patients to connect and engage with their health services online to access tools and resources to manage their health. In this paper, our PCHMS integrated an untethered PHR with well-being journeys, social forums, polls, diaries, and online messaging links with a health service provider.

However, a PCHMS often consists of multiple features, which refer to the functionalities available on the system. What are the features in a PCHMS that encourage consumers and patients to seek help or engage with health services for their well-being concerns? To date, it is still unclear what features contribute to an engaging and efficacious PCHMS.

Past studies have resulted in guidelines for the development of Internet interventions for consumer health [9-12]. Other studies have found features such as personalization, tailoring, and behavior feedback associated with significant consumer health behaviors when applied in the right context [13,14]. Researchers have also advocated for the use of behavioral theories, such as the health belief model (HBM) [15], social cognitive theory (SCT) [16], transtheoretical model (TTM) [17], and the theory of reasoned action / planned behavior [18], in the development of eHealth applications to increase their acceptability and efficacy. Yet, there is currently little literature to guide the features of PCHMS.

In parallel, the idea of creating a “bundle” of actions has recently been advocated as a way to address system inertia to change [19]. While its clinical applications have been shown to improve the quality and safety in managing ventilation-assisted pneumonia [20] and sepsis in intensive care [21], its applicability in eHealth has not been examined previously. A care bundle is a grouping of care elements for a particular symptom, procedure, or treatment [22]. It follows the holistic principle where a bundle, as a grouping of several evidence-based practices, when used in combination or as a cluster, should have a greater effect on the positive outcome of patients [22]. In eHealth, while evidence is emerging on which “individual” features are associated with significant consumer health behaviors, the concept of identifying a “bundle” of effective features in eHealth interventions has not been addressed previously.

For this reason, identifying features (or “bundles of features”) in a PCHMS that are associated with changes in consumers’ health behaviors remains a crucial area for research. In response, we designed an online prospective study to examine how a group of participants in a university setting used a PCHMS to manage their physical and emotional well-being. University students are known to experience elevated distress levels over an academic semester [3,23-30]. Yet, they are infrequent users of health services and hardly engage with services for assistance [31-33]. The aim of this study is to (1) examine whether use of a PCHMS is associated with increased rates of health service utilization and help-seeking behaviors for physical and/or emotional well-being, and (2) identify whether use of any specific PCHMS feature (ie, journey, personal health record, forum, poll, diary, or online appointment service), or bundles of features, is associated with help-seeking behaviors and health service utilization for well-being matters.

## Methods

### Trial Design and Participants

A one-group pre/posttest online prospective study was conducted over a university academic semester (July to November 2011). Inclusion criteria were (1) aged 18 or above, and (2) with access to the Internet and email at least on a monthly basis.

#### Study Protocol

Students and staff were approached via email lists and advertisements in online print publications, which described the study and invited interested parties to use a PCHMS called *Healthy.me* developed at the University of New South Wales (UNSW) to manage their physical and emotional well-being for an academic semester. Written informed consent was sought online from each participant. Participants then completed a 15-minute online pre-study survey, followed by a 5-minute mandatory online tutorial about *Healthy.me* prior to using the site. At study completion (end of semester), participants received an email asking them to complete a 15-minute online post-study survey. Two follow-up emails 5 days apart were sent as reminders to noncompleters. Those who completed all surveys were entered into a draw for an AU$500 gift voucher. A researcher was available via a dedicated telephone line and email to answer participants’ questions and concerns during the study. Ethics approval was obtained from the UNSW ethics committee.

#### Measures

At baseline, demographic information (such as age and gender) was collected, as well as information about their use of social networking websites, use of the Internet to find health-related information, and visits to a health professional (including whether they visited prior to the study a health care professional, University Health Service, and the University Counselling and Psychological Services).

In the pre- and post-study questionnaires, measures 1-3 were administered and additional measures (4-5) were administered in the postintervention questionnaire: (more details on each measure are available in Multimedia Appendix 1):

1. *COOP/WONCA charts* were used to evaluate participants’ functional status, defined as physical, emotional, and social status. These scales, which have been demonstrated to be a valid and feasible one-time screening assessment for mental disorders in primary care [34], measure six domains, namely physical fitness, feelings, daily activities, social activities, change in health, and overall health. Responses are via a 1-5 Likert-scale where higher scores indicate a poorer functional status.
2. *Well-being self-ratings and lifestyle intention*: adapted from the last question in the standardized instrument EUROQOL (EQ-5D) [35]*,* which measures health status, participants were asked to rate their physical and emotional well-being on a scale from 0 to 100. They were also asked to select one of four statements that best describes their intention to practice a lifestyle that benefits their well-being according to the transtheoretical model of behavior change [17].
3. *Health advice-seeking and health advice-providing networks*: adapted from the Norbeck Social Support Questionnaire [36], participants were asked to nominate up to 5 people they have sought advice from, or provided advice to, before and during the study.
4. *Help-seeking behaviors and health service utilization*: Help-seeking is defined as the behavior of actively seeking assistance [37], regardless of whether the source is informal or formal. A new scale was developed by the authors, adapted from the Actual Help-seeking Questionnaire (AHSQ) [37]. The scale covers help-seeking behaviors for physical and emotional well-being, informal and formal sources, as well as for self or others.
5. *Feedback on Healthy.me*: participants were asked to provide feedback on their overall experience of using *Healthy.me*, as well as their feedback on specific features on the website, using a range of scale items such as Likert scale, free-text comments, and checkbox answer options.

This paper focuses on usage of PCHMS features with consumers’ health behaviors and thus only reports participants’ help-seeking behaviors and health service utilization rates collected at post-study.

#### PCHMS Usage Metrics

A recent review by Danaher and Seeley [38] concluded there is no single, universally accepted measure for website usage, and researchers are still debating the best methods for defining and measuring website engagement [38].

In this study, we used simple website engagement measures to track participants’ activity on the website (ie, PCHMS login frequency and whether participants accessed, or did not access, each website feature). These measures were used to assess whether (1) there was a dose-response effect, that is, was the frequency of PCHMS login associated with rates of health service utilization and help-seeking behaviors, and whether (2) access to PCHMS feature(s) (ie, journey, personal health record, forum, poll, diary, and/or online appointment service) was associated with participants’ health service utilization and help-seeking behaviors for physical and/or emotional well-being.

PCHMS Web logs were analyzed to determine whether participants accessed (or did not access) any of the features at any time during the study. Some of these website engagement measures have previously been used to measure user engagement of PHR systems [39].

### Intervention

#### Theoretical Construct

The dose-response phenomenon tested in this study is related to the *familiarity principle*, *reinforcement effect*, and the *mere exposure effect* described by Zajonc [40], where the level of repeated exposure to an intervention is associated with participants developing a familiarity and preference for the intervention and thus increasing the likelihood to use it at times of need. Features such as length of exposure, the spread of experiences, the partitioning of episodes, the peak-and-end events in an incident, and the degradation or improvement in experience over time have been reported to influence a person’s overall impression of an experience [41]. While exposure to a website can be described using different measures, such as number of logins, repeated visits, and duration of visits, we used number of logins as our primary measure since it is one of the most common measures to describe participants’ engagement with a website.

#### Healthy.me

**Figure 1:**
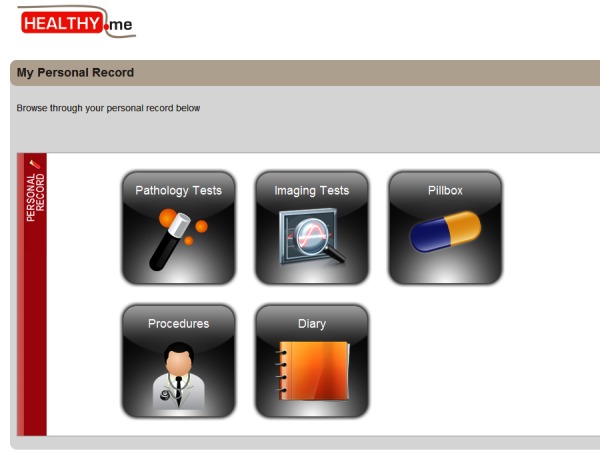
Personal Health Record on Healthy.me (University of New South Wales, 2009-2013).

**Figure 2:**
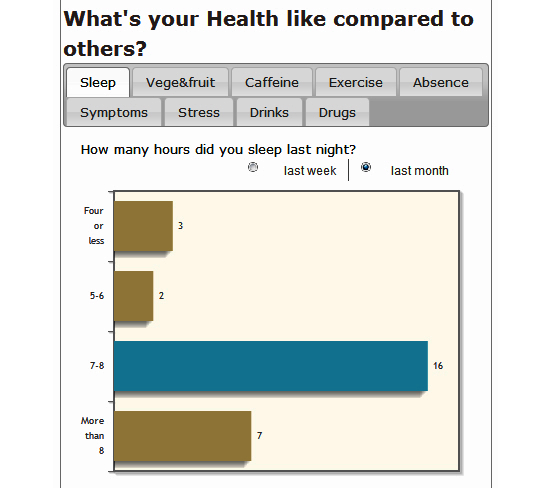
Poll on Healthy.me (University of New South Wales, 2009-2013).

*Healthy.me* was iteratively developed, and its first version was tested in other settings such as in vitro fertilization and influenza vaccination [42,43]. The first version contained features such as journey, the personal health record, and online appointment booking with the university primary care service. The version of *Healthy.me* (version 2.0) that was used in this study contained the above-mentioned features as well as online appointment booking with the university primary care and counselling services, a diary, forum, and poll. Details of each feature are described below:

1. Personal Health Record (PHR) for self-recording of medical test results, medications, scheduled appointments, and personnel looking after one’s health (see Figure 1).
2. Online appointment booking with the University Health Service (primary care) and the UNSW Counselling and Psychological Services (sent via email using the “Book now” button in the PCHMS).
3. Diary for participants to write down their thoughts about their health. By default, the diary is private. However, participants can select to share their diary with all participants enrolled in the PCHMS.
4. Social communication spaces, which support interaction across the continuum of care between fellow participants and clinicians. Features include the poll system and forums moderated by clinicians. Poll system in which participants answer simple health questions (eg, how much sleep did you get last night?), where they can view and compare their response with other participants’ aggregated answers in graph format (Figure 2). Forums moderated by clinicians (a primary care physician and a psychologist), where participants can either post their entries on the forum or send one-on-one email messages to other participants in the PCHMS (including clinicians). Guidelines on forum use and the protocol for responding to concerns reported in the forum were approved from the UNSW ethics committee. Posts sent by participants to the “Report concern” feature on the forum were emailed to clinical and research personnel during the study, who investigated any reported concerns. A Uniform Resource Locator (URL) available in the email to the dedicated staff allowed them to withdraw the forum post. The primary care physician and the psychologist not only moderated the forum but were also available to answer questions posted on the forums. No harm from the use of the forum or the PCHMS was reported by participants during the study.
5. Journeys that provide information for consumer participants to engage with clinicians and health services in an actionable way. Participants in this study had access to four well-being journeys for physical and emotional well-being: “Stay Healthy”, “Stressed out?”, “Feeling Anxious about the Exams?”, and “My Emotional Well-being Program”.

The four well-being journeys for physical and emotional well-being were designed and developed in consultation with University Counselling and Psychological Services psychologists and University Health Service primary care physicians, utilizing evidence-based consumer education material routinely used at UNSW to promote physical and emotional well-being. Written in youth-friendly language, using evidence-based mental health, psychoeducational, and psychosocial material, the journeys consisted of skills-focused content delivered online, as well as well-being workshops that participants could attend in-person at the University Counselling and Psychological Services. Participants could learn about mindfulness meditation, anxiety management, time management, and stress management at these workshops.

Journeys were delivered via the PCHMS at four pivotal time-points during a university academic semester (ie, beginning of semester, 4 weeks into semester, after mid-semester break, and before exams) to address physical and emotional well-being concerns likely to be concerning participants at each time-point. Participants were alerted with an email when a new journey became available on the PCHMS. These journeys provided task specific knowledge in an actionable way. For example, as participants read the journey for advice on physical or emotional well-being, they could immediately:

- book an appointment with a university primary care physician or a psychologist from the journey page,
- register to attend a well-being workshop,
- post a question on a forum to seek advice from fellow participants or a clinician (primary care physician or a psychologist), or
- send themselves an email reminder to do so later.

A pilot study was conducted in a controlled setting with 15 university staff and students of different ages, gender, and familiarity with computers to test the intervention, the measures, and the research design. Substantive usability issues were resolved before recruiting participants in their real-life setting.

### Data Analysis

Analysis was conducted on an intention-to-treat basis. Sequential logistic regression analyses were undertaken to prospectively examine the crude and adjusted odds ratios (ORs) for participants’ health service utilization and help-seeking behaviors for physical and emotional well-being matters [44]. Independent variables assessed included whether participants accessed (or did not access) each specific PCHMS feature (journey, personal health record, forum, poll, diary, and online appointment service), controlling for participant’s gender, age, and potential confounders (eg, whether the participant was a university service patient/client prior to the study) to provide a stratified estimate of intervention effect. The Pearson product-moment correlation coefficient was used to examine correlations among usage of features that were associated with consumers’ behaviors.

Participants’ health service utilization rates (ie, visits to a health professional, University Health Service, or the University Counselling and Psychological Services), and their help-seeking behaviors for physical or emotional well-being matters were compared at different PCHMS login frequency thresholds (zero logins, once only, two to five times, six to 10 times, more than 10 times). The rationale for selecting these login frequency cutoffs is based on using heuristics to ensure important login frequency thresholds are covered (ie, zero, once only, and ≥ a high login frequency threshold) and that there are sufficient data points in each frequency threshold to conduct analyses.

Between group analyses were conducted using chi-square analysis. Participants’ pre-study characteristics (namely use of the Internet to find health information, use of social networking websites, visits to a health care professional in the past 6 months, and their self-rated well-being ratings classified as over or below 50 at pre-study) were compared between different PCHMS login frequencies using chi-square to assess whether these characteristics were associated with PCHMS usage levels. Descriptive analyses were conducted on participants’ reasons for *not* seeking help during study.

Data analysis was performed using IBM SPSS Statistics 20 [45]. Tests performed were two-tailed and assumed a cutoff of *P*<.05 for statistical significance.

## Results

### Participants

**Figure 3:**
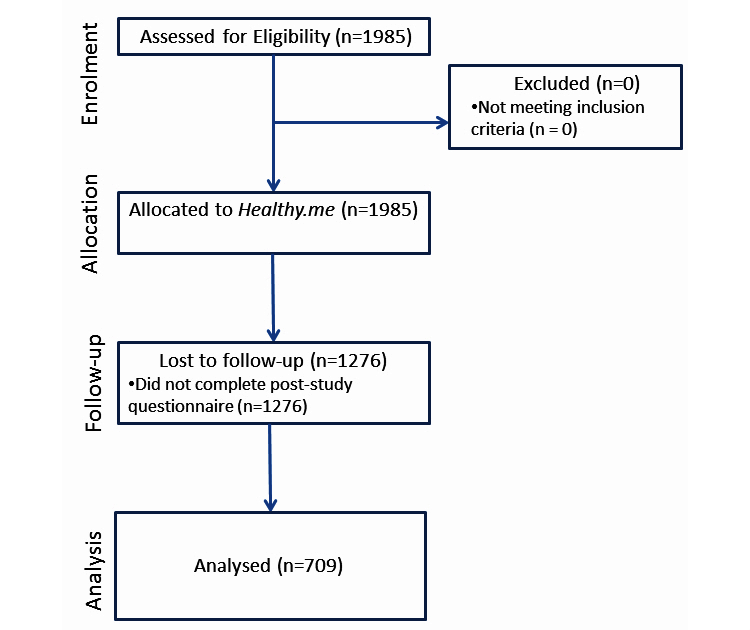
Participant flowchart in the study.

A total of 1985 participants met inclusion criteria and were recruited into the study. All completed the pre-study questionnaire. Of those, 709 completed the post-study questionnaire (Figure 3). Analyses were conducted on 709 eligible participants who completed both the pre-study and post-study questionnaires. Of these, 81% (572/709) participants logged into the PCHMS at least once. No significant differences were found between questionnaires completers and noncompleters in their pre-study characteristics or the number of PCHMS login sessions (*P*>.05). Among questionnaire completers, no significant differences were found between different PCHMS login frequencies (*P*>.05).

Baseline characteristics of eligible participants are presented in Table 1. Participants’ well-being concerns during the study, their help-seeking behaviors, and their purpose for visiting a health care professional are outlined in Table 2.

### Health Service Utilization

**Figure 4:**
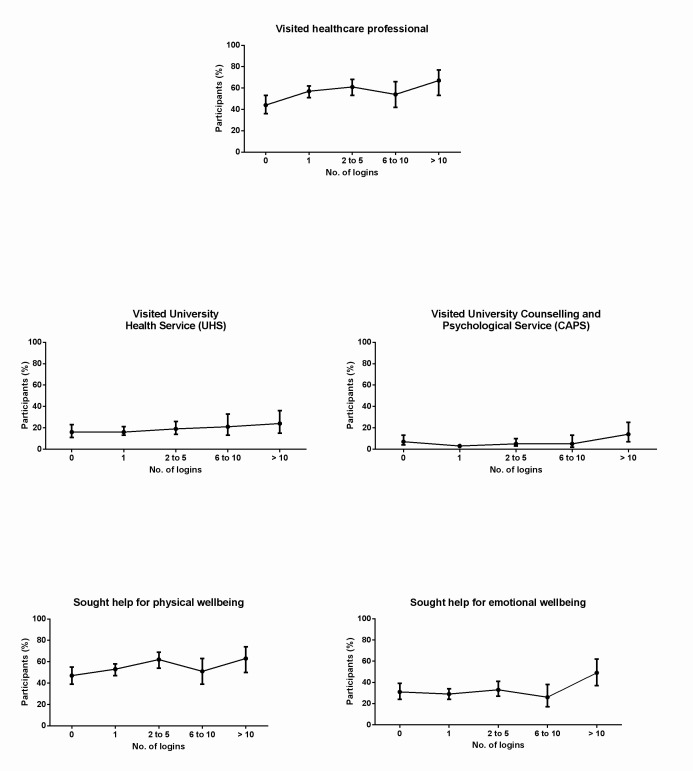
Health service utilization rates and help-seeking behaviors between different PCHMS login frequency thresholds.

Overall, 50% (358/709) of participants visited a health care professional (for themselves or others) for a physical well-being concern and 13% (95/709) for emotional well-being during the study (Table 2). Health service utilization rates (ie, visits to a health professional, University Health Service, and University Counselling and Psychological Services) during the study are outlined in Table 3 and Figure 4 according to PCHMS login frequency.

**Table 1:** Baseline characteristics of study participants who completed both pre-study and post-study questionnaires.

Characteristics		Total n=709 (%)
Mean age, years (SD)		25.2 (9.41)
Female gender (%)		427 (60.2%)
University student		625 (88.1%)
Non-medicine faculty ^a^		570 (80.4%)
Patient at University Health Service (prior to study)		148 (20.9%)
Visited UNSW Counselling and Psychological Service (prior to study)		83 (11.7%)
**Use of social networking websites**		
	Several times a day	434 (61.2%)
	Several times a week	183 (25.8%)
	Several times a month	29 (4.1%)
	Less often	39 (5.5%)
	I do not use social networking websites	24 (3.4%)
**Use of Internet to find health-related information**		
	Several times a week	79 (11.1%)
	Few times a month	161 (22.7%)
	Less often	93 (13.1%)
	Never	38 (5.4%)
**Visited health care professional(s) in past 6 months**		
	None	188 (26.5%)
	Once only	173 (24.4%)
	Two to three times	238 (33.6%)
	More often	110 (15.5%)

^a^Faculty refers to the School or the Faculty that a participant is from, regardless of whether he/she is a student or a staff member.

**Table 2:** Participants’ health service utilization, help-seeking behaviors, and experiences of physical and emotional well-being concerns during the study.

		Number n=709 (%)
**Self-experience well-being concern**		
	I experienced a physical well-being concern during study	479 (67.6%)
	I experienced an emotional well-being concern during study	422 (59.5%)
**Encountered someone with well-being concerns**		
	I encountered someone with physical well-being concerns during study	400 (56.4%)
	I encountered someone with well-being concerns during study	365 (51.5%)
**Health service utilization**		
	I visited a health care professional for only physical well-being concerns (for self or others)	276 (38.9%)
	I visited a health care professional for only emotional well-being concerns (for self or others)	13 (1.8%)
	I visited a health care professional for both physical and emotional well-being concerns (for self or others)	82 (11.6%)
**Help seeking (formal or informal sources)**		
	I sought advice on physical well-being (for myself)	370 (52.2%)
	I sought advice on physical well-being (for others)	88 (12.4%)
	I sought advice on emotional well-being (for myself)	201 (28.3%)
	I sought advice on emotional well-being (for others)	75 (10.6%)
**Not seeking or providing help**		
	There was a need for physical well-being assistance (for self or others), but I did not seek help	109 (15.4%)
	There was a need for emotional well-being assistance (for self or others), but I did not seek help	221 (31.2%)
**Confidence in providing help to others on, mean (SD)** ^a^		
	Physical well-being	2.2 (0.87)
	Emotional well-being	2.2 (0.82)

^a^Confidence: 1=not confident, 2=quite confident, 3=confident, 4=very confident

**Table 3:** Health service utilization and help-seeking behaviors according to different usage levels of PCHMS.

	% (95% CI)				
No. of PCHMS logins ^a^	Visited health professional ^b^	Visited University Health Service ^c^	Visited University Counselling and Psychological Services ^d^	Sought help for physical well-being ^e^	Sought help for emotional well-being ^f^
0 (n=136)	44 (36 to 53)	16 (11 to 23)	7 (4 to 13)	47 (39 to 55)	31 (24 to 39)
1 (n=287)	57 (51 to 62)	16 (13 to 21)	4 (2 to 6)	53 (47 to 58)	29 (24 to 34)
2 to 5 (n=165)	61 (53 to 68)	19 (14 to 26)	6 (3 to 10)	62 (54 to 69)	33 (27 to 41)
6 to 10 (n=61)	54 (42 to 66)	21 (13 to 33)	5 (2 to 13)	51 (39 to 63)	26 (17 to 38)
≥ 10 (n=59)	67 (53 to 77)	24 (15 to 36)	14 (7 to 25)	63 (50 to 74)	49 (37 to 62)

^a^1 participant was excluded as his/her no. of logins is recorded as >4000. Among the 708 participants included in this analysis, the mean of login frequency is 4.3, standard deviation is 19.05, and the maximum number of logins is 456.

^b^Visited health professional during study: χ^2^_4_=11.80, *P*=.019, n=708.

^c^Visited University Health Service during study: χ^2^_4_=2.79, *P*=.59, n=708.

^d^Visited UNSW Counselling and Psychological Service during study: χ^2^_4_=10.26, *P*=.036, n=708.

^e^Sought help for physical well-being during study: χ^2^_4_=8.94, *P*=.063, n=708.

^f^Sought help for emotional well-being during study: χ^2^_4_=10.70, *P*=.03, n=708.

In absolute terms, participants who logged into the PCHMS more than 10 times were 22 percentage points more than those who never logged in to visit a health care professional during the study: χ^2^_4_=11.80, *P*=.01, n=708; zero logins: 44% (60/136, 95% CI 36-53) vs ≥10 logins: 66% (39/59, 95% CI 53-77). Relative to participants who never logged in to the PCHMS, the proportion of participants visiting a health care professional during study was 50% higher for those who logged into the PCHMS more than 10 times: RR is 1.50 (95% CI 1.15-1.95).

Visits to the University Health Service did not differ significantly between different PCHMS login frequency thresholds: χ^2^_4_=2.79, *P*=.59, n=708. However, participants who logged into the PCHMS more than 10 times were 10 percentage points more than less-engaged PCHMS users to visit the University Counselling and Psychological Services during the study: χ^2^_4_=10.26, *P*=.03, n=708; once only was 4% (10/287, 95% CI 2-6) vs ≥ 10 logins at 14% (8/59, 95% CI 7-25). Relative to participants who logged into the PCHMS only once, the proportion of participants visiting the University Counselling and Psychological Services during the study was 289% higher for those who logged into the PCHMS more than 10 times: RR is 3.85 (95% CI 1.60-9.44).

### Help-Seeking Behaviors

Overall, 54% (386/709) of participants sought formal or informal help (for themselves or others) for physical well-being and 32% (225/709) for emotional well-being during the study (Table 2). Participants’ help-seeking behaviors during the study between different PCHMS login frequency thresholds are described in Table 3 and Figure 4.

Help-seeking behaviors for physical well-being matters did not differ significantly between different PCHMS login frequencies: χ^2^_4_=8.94, *P*=.06, n=708. However, participants who logged into the PCHMS 10 times or more were 20 percentage points more likely to seek help for an emotional well-being matter than less-engaged users, ie, χ^2^_4_=10.70, *P*=.03, n=708; once only: 29% (82/287, 95% CI 24-34) vs > 10 logins: 49% (29/59, 95% CI 37-62). Relative to participants who logged into the PCHMS only once, the proportion of participants seeking assistance for an emotional well-being matter during the study was 72% higher for those who logged into the PCHMS more than 10 times, ie, RR is 1.72 (95% CI 1.25-2.36).

#### Reasons for Not Seeking Help

Reasons for *not* seeking help during the study are outlined in Tables 4 and 5. The most frequent reason for not seeking help for a physical or emotional well-being matter was “no time / inconvenience”, that is, physical: 51.4% (56/109); and emotional: 42.5% (94/221). Among those who did not seek help for their *emotional* well-being concern (n=221), the next frequently cited reason was “fear of confrontation and learning about the health issue” (40.0%), followed by “I didn’t think anyone (or anything) could help” (36.2%). Among those who did not seek help for their *physical* well-being concern (n=109), the second most frequently cited reason was “I didn’t know (or still don’t know) what seems to be the problem” (31.2%), followed by “cost” (29.4%).

#### Feature Bundles Associated With Health Service Utilization and Help-Seeking Behaviors

Different groups of system features were correlated with different consumer behaviors:

- *Health service utilization* was strongly correlated with use of a bundle of features involving *online appointment booking* (primary care: OR 1.74, 95% CI 1.01-3.00; counselling: OR 6.04, 95% CI 2.30-15.85), *personal health record* (health care professional: OR 2.82, 95% CI 1.63-4.89), the *poll* (health care professional: OR 1.47, 95% CI 1.02-2.12), and *diary* (counselling: OR 4.92, 95% CI 1.40-17.35). For participants who utilized a health service, there was a strong positive correlation in usage frequency between the diary and poll (*r*=0.726, n=424, *P*<.001), a moderate positive correlation between the personal health record and poll (*r*=0.321, n=424, *P*<.001), and a small positive correlation between the personal health record and online appointment booking (*r*=0.234, n=424, *P*<.001).
- Formal or informal help-seeking behaviors for *physical* well-being matters was only correlated with use of the *personal health record* (OR 1.73, 95% CI 1.18-2.53).
- Help-seeking for *emotional* well-being concerns (including visits to the university counselling service) was correlated with a bundle comprising the *poll* (formal or informal help-seeking: OR 1.03, 95% CI 1.00-1.05), *diary* (counselling: OR 4.92, 95% CI 1.40-17.35), and *online appointment booking* (counselling: OR 6.04, 95% CI 2.30-15.85). For participants who sought help for emotional well-being concerns and/or visited the university counselling service, there was a strong positive correlation in usage frequency between the diary and poll (*r*=0.787, n=230, *P*<.001), and a small positive correlation between the poll and online appointment booking (*r*=0.145, n=230, *P*=.028).

**Figure 5:**
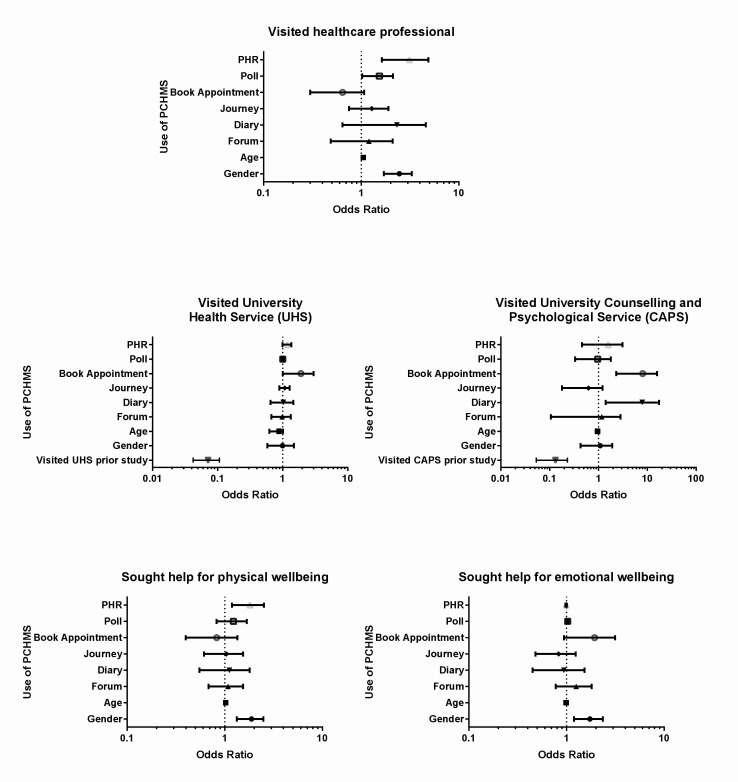
PCHMS features associated with health service utilization and help-seeking behaviors.

Full details of the logistic regression models are summarized in Figure 5 and Multimedia Appendix 2.

**Table 4:** Reasons for *not* seeking help for physical well-being concerns during study (n=109).

Reason	Number ^a^ n=109 (%)
No time / inconvenience	56 (51.4%)
I didn’t know (or still don’t know) what seems to be the problem	34 (31.2%)
Cost	32 (29.4%)
Not well enough (or motivated) to seek help	27 (24.8%)
I didn’t think anyone (or anything) can help	22 (20.2%)
I didn’t know how to seek help	16 (14.7%)
Fear of what others may think	14 (12.8%)
Fear of confrontation and learning about the health issue	14 (12.8%)
Previous unsatisfactory contacts with health care professionals	9 (8.3%)
Stigma or cultural attitudes	8 (7.3%)
Other	7 (6.4%)

^a^Participants who experienced a physical well-being concern during study but did not seek help. Participants can select more than one reason.

**Table 5:** Reasons for *not* seeking help for emotional well-being concerns during study (n=221).

Reason	Number ^a^ n=221 (%)
No time / inconvenience	94 (42.5%)
Fear of confrontation and learning about the health issue	31 (40.0%)
I didn’t think anyone (or anything) can help	80 (36.2%)
I didn’t know (or still don’t know) what seems to be the problem	59 (26.7%)
Not well enough (or motivated) to seek help	54 (24.4%)
Cost	46 (20.8%)
Fear of what others may think	46 (20.8%)
I didn’t know how to seek help	39 (17.6%)
Stigma or cultural attitudes	33 (14.9%)
Other	27 (12.2%)
Previous unsatisfactory contacts with health care professionals	19 (8.6%)

^a^Participants who experienced an emotional well-being concern during study but did not seek help. Participants can select more than one reason.

## Discussion

### Dose-Response Effect

This is the first study that shows a dose-response effect of using a PCHMS on consumers’ health service utilization (primary care and counselling) and help-seeking behaviors for emotional well-being. To our knowledge, this is also the first study that contributes an understanding of which bundles of PCHMS features are associated with consumer help-seeking and health service utilization behaviors.

The nature of this study only allows associational inferences to be drawn and specifically, we cannot say that it was the usage of these features that drove user behaviors. An alternate reading of our results is that those individuals who are most likely to use health services are also the population most likely to be drawn to use a PCHMS. Both causal readings are of interest, and it is also likely that both probably are to some extent in operation in the results reported here. As the pre-study characteristics and well-being ratings of users were uniformly distributed across different PCHMS login frequency thresholds, we could not in this study detect differences in users to explain differences in their behaviors, suggesting the dose-response reading of our results is more likely the explanation. Untangling these two alternate readings will undoubtedly be resolved with further research.

The dose-response effect observed in this study may be explained by the availability heuristic, which describes the “situation in which people assess the frequency of a class or the probability of an event by the ease with which instances or occurrences can be brought to mind.” [46]. At times when participants experienced emotional and/or physical well-being matters, they may have utilized the availability heuristic to recall mechanisms they have been exposed to (in this case possibly the PCHMS), and accessed it to seek help and/or engage with health services. In addition, by providing informational cues in the PCHMS that are directly linked to an action (such as a “Book now” button embedded within consumer specific content) [15,43], we may have helped participants overcome their perceived barriers to visiting a health service (such as no time, inconvenience, or having to explain one’s emotional concern over the phone), which may have hindered the step in making an appointment. Moreover, the PCHMS may have provided a supplementary tool for participants who are already patients at participating health services to manage their health care concerns in conjunction with the services.

### Feature Bundles

A PCHMS in practice offers a “bundle” of eHealth services and features including but not limited to a PHR. However, no studies to date have examined which bundles of features might motivate consumers’ health behaviors. Past studies that examined user engagement investigated the whole website [47-49], but not individual or bundles of features and not on associating PCHMS engagement with health behaviors.

While help-seeking for physical well-being was only correlated with use of the personal health record, a bundle of PCHMS features were correlated with emotional well-being help-seeking. Providing an environment that allows self-reflection (diary), social feedback (poll), and reducing the barriers to engage with health services (online appointment booking) appeared to work in combination for emotional well-being help-seeking. As suggested by Coiera, one reason such bundles might work is that they are programmatic, bringing together components that reinforce each other’s value and use [19]. Unpacking the use and impact of complex eHealth interventions from a “bundle” perspective should help us understand the right type, number, and complexity of features needed for consumers’ health behaviors. Identifying effective feature bundles thus appears to be a logical principle to follow in designing the next generation of PCHMS and PHR-related systems.

### Feature Rationale

There are strong theoretical reasons why the features tested in this study could drive behavioral change:

- *The online appointment booking service,* embedded within health service information descriptions (ie, journeys), allows consumers to turn information into action. Use of the feature was significantly associated with visits to university primary care and counselling services, in keeping with the “cue to action” elements of the HBM [15].
- *Personal health records,* which encouraged participants to keep track of their personal health details (such as medication, test results, scheduled appointments, or health care team members), were significantly associated with visits to a health care professional and help-seeking for physical well-being matters. This is related to increasing one’s self-efficacy by being aware of past and upcoming tasks and results [50].
- *A diary,* which encouraged self-reflection and self-awareness was also significantly associated with visits to the university counselling service, in accord with the principle of self-monitoring—one of the most common behavioral change techniques [51].
- *The poll,* which provided social feedback and social connectivity was also significantly associated with visits to a health care professional and help-seeking for emotional well-being matters. This is congruent with SCT [16] and the “subjective norms” aspect in the Theory of Planned Behavior / Reasoned Action [18], where an individual’s perception of social normative pressures affects whether one will conduct such behavior. Social networking features that provide social norm information or allow participants to “discover” and connect with similar individuals may lead to greater engagement with the PCHMS.

### Comparison With Prior Work

Our findings are in line with the emerging body of literature that associates eHealth interventions with consumers’ health behaviors (such as personalization, tailoring, and behavioral feedback [13,14,51,52]). Previously, we have demonstrated that these PCHMS features have broad utility in a number of health areas including in vitro fertilization and influenza vaccination [42,43]. The current study extends those applications to help-seeking behaviors for physical and emotional well-being.

Our findings also extend previous studies that describe models, guidelines, and definitions on Internet interventions for consumers’ health behaviors [9-12], especially PHR-related systems. For PCHMS to be valued by consumers, clinicians, and health service providers, they need to (1) address pressing needs faced by patients and consumers, (2) enable patients and consumers to accomplish tasks without further complicating their lives, and (3) avoid unnecessarily disrupting clinicians’ workload or increasing pressure on health service providers [13].

Attrition is a significant concern in consumer eHealth research [5,53]. The Internet has the capacity to reach many individuals who may never seek formal treatment at physical or mental health services. Stumbling across an eHealth application, even for a short period of time, may trigger the much-needed opportunity for those in need to reflect and seek appropriate help. As suggested by Christensen and Mackinnon, the primary role of the Internet in disease prevention and early intervention could possibly be in the delivery of short positive health messages [53]. This study has identified bundles of features in a PCHMS that are associated with consumers’ help-seeking and health service utilization rates. Encouraging participants to engage in “relevant” features, rather than the whole intervention, at times of need may lead to the desired health behaviors and outcomes. In fact, website adherence or “stickiness” may cease to be an issue when “sufficient” engagement with the “right” bundle of features are demonstrated to lead to similar health outcomes and help-seeking behaviors [53].

### Strengths and Limitations

Key strengths include the large number of participants, a multifaceted PCHMS with connectivity to health service providers that model many of the generic PHR systems and the use of PCHMS usage metrics to associate with consumers’ health behaviors. Some limitations include:

1. University setting: Participants in a university setting may have been more motivated and willing to try new technologies to manage their health than the general population [38,49]. An additional limitation is the short duration of the study (5 months). High attrition rates are common in eHealth intervention studies, with a recent systematic review revealing that completion of protocol rates for depression sites ranged from 43% to 99% [54]. One of the possible reasons for the attrition rate of 64% in this study is that participants were asked by email to complete their post-study questionnaire during the long university summer break, where students and staff were not as likely to check their university email. However, the number of participants eligible for analysis is still relatively large in this study (ie, 709), with 81% logging into the PCHMS at least once, providing a sufficient sample size to analyze whether participants’ usage of the PCHMS is associated with their health service and help-seeking utilization rates. Overall, future studies conducted in the university setting should avoid commencing or completing the study during university breaks.
2. Self-reports and self-entry functionality: The study relied on self-reports by participants, which have been shown to be acceptable in studies of help-seeking, health service utilization, and mental health-related studies among students [30,55-57]. The PCHMS currently relies on self-entry functionality, which may have caused lower usage of the tool and reduced follow-up data collection. While it is possible that some patients could have used the PCHMS after visiting the university health services, we validated health service utilization rates by matching self-report from a subset of study participants with their health records at the University Counselling and Psychological Services, where system usage log files indicated that usage of the PCHMS preceded clinic visits or that the “Book now” button was used. Although this study focuses only on eligible participants who completed both the pre- and post-study questionnaires, this approach is appropriate as our aim is to identify whether help-seeking and health service utilization behaviors (collected only at post-study) are associated with participants’ usage of the PCHMS. In fact, this study was conducted using intention-to-treat analysis as all eligible participants who used or did not use the PCHMS were included in the analysis.
3. Causality vs association: Although findings in this study are limited by its cross-sectional nature and we could attribute no causal relationships, our findings concur with Couper and colleagues’ study, which found that website engagement was significantly associated with consumers’ health behaviors [47]. In addition, our analyses showed that participants’ pre-study characteristics and well-being ratings were uniformly distributed between different PCHMS login frequency thresholds.
4. PCHMS engagement measures: studies have reported numerous metrics for measuring user engagement with a website, such as number of website visits, time spent on a site, and number of features used [38,58]. This study used simple website engagement measures. Future studies could consider in-depth analyses of whether participants accessed a bundle of features in the same login session, or whether the features were accessed in a particular sequence over the duration of the study. In addition, future studies should consider incorporating a qualitative component to elicit participants’ context and reasons (eg, why and how) for engaging with the website.

### Conclusions

Our online prospective study provides evidence that PCHMS usage is associated with consumers’ utilization of health services and help-seeking behaviors for emotional well-being concerns. The features in this PCHMS are sufficiently general to be applicable to a variety of help-seeking and preventative health tasks.

While there is evidence that Web interventions can trigger significant consumer health behaviors, the empirical and theoretical basis for developing PCHMS features in general is still weak. Abandoning an eHealth application is a common and significant phenomenon. Asking participants to engage in *all* features of an eHealth application may not be an effective strategy to reduce attrition or influence health behaviors. Strategies that alert participants to bundles of features that are of immediate relevance or benefit to them could possibly result in higher engagement with the intervention and achieve the desired health actions. Future studies should investigate whether participants’ engagement with “tailored” bundles of PCHMS features are effective in achieving the desired health behaviors and outcomes, and that more correlation studies are needed to show which bundles of features are best for which kinds of consumers’ health tasks, conditions, and help-seeking stages.

## Multimedia Appendix 1

Details on questionnaire measures.

## Multimedia Appendix 2

Details of logistic regression results for each outcome measure.

## Abbreviations

HBM: health belief model
OR: odds ratio
PCHMS: personally controlled health management system
PHR: personal health record
RCT: randomized controlled trial
RR: relative risk
SCT: social cognitive theory
TTM: transtheoretical model
UNSW: University of New South Wales

## References

[1] Greenhalgh T, Stramer K, Bratan T, Byrne E, Russell J, Potts HW (2010). Adoption and non-adoption of a shared electronic summary record in England: a mixed-method case study. BMJ.

[2] Greenhalgh T, Hinder S, Stramer K, Bratan T, Russell J (2010). Adoption, non-adoption, and abandonment of a personal electronic health record: case study of HealthSpace. BMJ.

[3] Black AD, Car J, Pagliari C, Anandan C, Cresswell K, Bokun T, McKinstry B, Procter R, Majeed A, Sheikh A (2011). The impact of eHealth on the quality and safety of health care: a systematic overview. PLoS Med.

[4] Khadjesari Z, Murray E, Kalaitzaki E, White IR, McCambridge J, Thompson SG, Wallace P, Godfrey C (2011). Impact and costs of incentives to reduce attrition in online trials: two randomized controlled trials. J Med Internet Res.

[5] Eysenbach G (2005). The law of attrition. J Med Internet Res.

[6] Poirier J, Cobb NK (2012). Social influence as a driver of engagement in a web-based health intervention. J Med Internet Res.

[7] Institute of Medicine (2011). Health IT and Patient Safety: Building Safer Systems for Better Care.

[8] Tang PC, Ash JS, Bates DW, Overhage JM, Sands DZ (2006). Personal health records: definitions, benefits, and strategies for overcoming barriers to adoption. J Am Med Inform Assoc.

[9] Ritterband LM, Thorndike FP, Cox DJ, Kovatchev BP, Gonder-Frederick LA (2009). A behavior change model for internet interventions. Ann Behav Med.

[10] Proudfoot J, Klein B, Barak A, Carlbring P, Cuijpers P, Lange A, Ritterband L, Andersson G (2011). Establishing Guidelines for Executing and Reporting Internet Intervention Research. Cognitive Behaviour Therapy.

[11] Barak A, Klein B, Proudfoot JG (2009). Defining internet-supported therapeutic interventions. Ann Behav Med.

[12] Krukowski RA, Harvey-Berino J, Ashikaga T, Thomas CS, Micco N (2008). Internet-based weight control: the relationship between web features and weight loss. Telemed J E Health.

[13] Gibbons C, Wilson RF, Samal L, Lehmann CU, Dickersin K, Lehmann HP (2009). The John Hopkins University Evidence-based Practice Centre.

[14] Noar SM, Benac CN, Harris MS (2007). Does tailoring matter? Meta-analytic review of tailored print health behavior change interventions. Psychol Bull.

[15] Janz NK, Becker MH (1984). The Health Belief Model: a decade later. Health Educ Q.

[16] Bandura A (1989). Human agency in social cognitive theory. Am Psychol.

[17] Prochaska J, DiClemente C (1984). The Transtheoretical Approach: Crossing the Traditional Boundaries of Therapy.

[18] Ajzen I (1991). The Theory of Planned Behavior. Organ Behav Hum Decis Process.

[19] Coiera E (2011). Why system inertia makes health reform so difficult. BMJ.

[20] Albertos R, Caralt B, Rello J (2011). Ventilator-associated pneumonia management in critical illness. Curr Opin Gastroenterol.

[21] Barochia AV, Cui X, Vitberg D, Suffredini AF, O'Grady NP, Banks SM, Minneci P, Kern SJ, Danner RL, Natanson C, Eichacker PQ (2010). Bundled care for septic shock: an analysis of clinical trials. Crit Care Med.

[22] Fulbrook P, Mooney S (2003). Care bundles in critical care: a practical approach to evidence-based practice. Nurs Crit Care.

[23] Stallman HM (2008). Prevalence of psychological distress in university students--implications for service delivery. Aust Fam Physician.

[24] Stallman HM, Shochet I (2009). Prevalence of mental health problems in Australian university health services. Australian Psychologist.

[25] Leahy CM, Peterson RF, Wilson IG, Newbury JW, Tonkin AL, Turnbull D (2010). Distress levels and self-reported treatment rates for medicine, law, psychology and mechanical engineering tertiary students: cross-sectional study. Aust N Z J Psychiatry.

[26] Stallman HM (2010). Psychological distress in university students: A comparison with general population data. Australian Psychologist.

[27] Song Y, Huang Y, Liu D, Kwan JS, Zhang F, Sham PC, Tang SW (2008). Depression in college: depressive symptoms and personality factors in Beijing and Hong Kong college freshmen. Compr Psychiatry.

[28] Gallagher R (2008). National survey of counselling center directors.

[29] Bayram N, Bilgel N (2008). The prevalence and socio-demographic correlations of depression, anxiety and stress among a group of university students. Soc Psychiatry Psychiatr Epidemiol.

[30] Eisenberg D, Golberstein E, Hunt J (2009). Mental Health and Academic Success in College. BE Journal of Economic Analysis & Policy.

[31] Ciarrochi J, Deane FP, Wilson CJ, Rickwood D (2002). Adolescents who need help the most are the least likely to seek it: the relationship between low emotional competence and low intention to seek help. British Journal of Guidance and Counselling.

[32] Yap MB, Reavley NJ, Jorm AF (2012). Australian youth still have limited awareness of headspace: results from a national survey. Aust N Z J Psychiatry.

[33] Britt HMG, Charles J, Henderson J, Bayram C, Valenti L, Pan Y, Harrison C, O'Halloran J, Fahridin S, Chambers T General practice activity in Australia 2000-01 to 2009-10.

[34] de Azevedo-Marques JM, Zuardi AW (2011). COOP/WONCA charts as a screen for mental disorders in primary care. Ann Fam Med.

[35] Rabin R, de Charro F (2001). EQ-5D: a measure of health status from the EuroQol Group. Ann Med.

[36] Norbeck JS (1984). The Norbeck Social Support Questionnaire. Birth Defects Orig Artic Ser.

[37] Rickwood D, Deane FP, Wilson CJ, Ciarrochi J (2005). Young people's help-seeking for mental health problems. Advances in Mental Health.

[38] Danaher BG, Seeley JR (2009). Methodological issues in research on web-based behavioral interventions. Ann Behav Med.

[39] Wicks P, Massagli M, Frost J, Brownstein C, Okun S, Vaughan T, Bradley R, Heywood J (2010). Sharing health data for better outcomes on PatientsLikeMe. J Med Internet Res.

[40] Zajonc RB (1968). The Attitudinal Effects of Mere Exposure. Journal of Personality and Social Psychology.

[41] Varey CA, Kahneman D (1992). Experiences extended across time: Evaluation of moments and episodes. J. Behav. Decis. Making.

[42] Lau AYS, Parker A, Early J, Sacks G, Anvari F, Coiera E (2012). Comparative usage of a web-based personally controlled health management systemnormal support: a case study in IVF. electronic Journal of Health Informatics (eJHI).

[43] Lau AY, Sintchenko V, Crimmins J, Magrabi F, Gallego B, Coiera E (2012). Impact of a web-based personally controlled health management system on influenza vaccination and health services utilization rates: a randomized controlled trial. J Am Med Inform Assoc.

[44] Tabachnick B, Fidell L (2006). Using Multivariate Statistics. 5th edn. ed.

[45] (2012). IBM.

[46] Kahneman D, Slovic P, Tversky A (1982). Judgment under uncertainty: Heuristics and biases.

[47] Couper MP, Alexander GL, Zhang N, Little RJ, Maddy N, Nowak MA, McClure JB, Calvi JJ, Rolnick SJ, Stopponi MA, Cole Johnson C (2010). Engagement and retention: measuring breadth and depth of participant use of an online intervention. J Med Internet Res.

[48] Glasgow ER, Christiansen MS, Kurz D, King KD, Woolley T, Faber JA, Estabrooks PA, Strycker L, Toobert D, Dickman J (2011). Engagement in a diabetes self-management website: usage patterns and generalizability of program use. J Med Internet Res.

[49] Glasgow RE, Nelson CC, Kearney KA, Reid R, Ritzwoller DP, Strecher VJ, Couper MP, Green B, Wildenhaus K (2007). Reach, engagement, and retention in an Internet-based weight loss program in a multi-site randomized controlled trial. J Med Internet Res.

[50] Bandura A (1977). Self-efficacy: toward a unifying theory of behavioral change. Psychol Rev.

[51] Webb TL, Joseph J, Yardley L, Michie S (2010). Using the internet to promote health behavior change: a systematic review and meta-analysis of the impact of theoretical basis, use of behavior change techniques, and mode of delivery on efficacy. J Med Internet Res.

[52] Kelders SM, Kok RN, Ossebaard HC, Van Gemert-Pijnen JE (2012). Persuasive system design does matter: a systematic review of adherence to web-based interventions. J Med Internet Res.

[53] Christensen H, Mackinnon A (2006). The law of attrition revisited. J Med Internet Res.

[54] Christensen H, Griffiths MK, Farrer L (2009). Adherence in internet interventions for anxiety and depression. J Med Internet Res.

[55] Eisenberg D, Downs MF, Golberstein E, Zivin K (2009). Stigma and help seeking for mental health among college students. Med Care Res Rev.

[56] Eisenberg D, Golberstein E, Gollust SE (2007). Help-seeking and access to mental health care in a university student population. Med Care.

[57] Eisenberg D, Gollust SE, Golberstein E, Hefner JL (2007). Prevalence and correlates of depression, anxiety, and suicidality among university students. Am J Orthopsychiatry.

[58] Strecher VJ, McClure JB, Alexander GL, Chakraborty B, Nair VN, Konkel JM, Greene SM, Collins LM, Carlier CC, Wiese CJ, Little RJ, Pomerleau CS, Pomerleau OF (2008). Web-based smoking-cessation programs: results of a randomized trial. Am J Prev Med.

