# Supplementary material for: Which Bundles of Features in a Web-Based Personally Controlled Health Management System Are Associated With Consumer Help-Seeking Behaviors for Physical and Emotional Well-Being?
Source: J Med Internet Res. 2013 May 6;15(5):e79. doi: 10.2196/jmir.2414 (PMC3650927; doi:10.2196/jmir.2414)
Supplement: Supplementary file 1 [file jmir_v15i5e79_app1.pdf]

Appendix 1. Details on questionnaire measures.

At baseline, demographic information (such as age and gender) was collected, as well as information about their use of social networking websites, use of the Internet to find health-related information, and visits to a health professional (including whether they visited prior to the study a healthcare professional, University Health Service, and the University Counselling and Psychological Services).

In the pre- and post-study questionnaires, the following measures were administered:

(1) *COOP/WONCA charts* were used to evaluate participants' functional status, defined as physical, emotional, and social status. These scales, which have been demonstrated to be a valid and feasible one-time screening assessment for mental disorders in primary care [33], measure six domains namely physical fitness, feelings, daily activities, social activities, change in health, and overall health. Responses are via a 1-5 Likert-scale where higher scores indicate a poorer functional status.

(2) *Well-being self-ratings and lifestyle intention*: adapted from the last question in the standardized instrument *EUROQOL (EQ-5D)* [34], which measures health status, participants were asked to rate their physical and emotional well-being on a scale from 0 to 100: "I would like you to think of a scale between 0 and 100 (where 0 is the worst state you can imagine and 100 is the best you can imagine). What number between 0 and 100 best describes your physical [emotional] well-being today?". They were also asked to select one of four statements that best describes their intention to practice a lifestyle that benefits their well-being according to the Transtheoretical Model of Behavior Change [16]. An example of a statement is "I intend to practice a lifestyle that would benefit my well-being in the next 6 months."

(3) *Health advice-seeking and health advice-providing networks*: adapted from *the Norbeck Social Support Questionnair*e [35], participants were asked to nominate up to five people they have sought advice from, or provided advice to, before and during the study. Here participants completed the following question: “Looking back over the past six months [or during the semester (Jul-Nov 2011)], who asked you for advice on a health issue? [or who did you ask for advice on a health issue?]”. For each nominated person, the participant indicated their i) relationship (partner, parent, sibling, other family member, friend, colleague, acquaintance, healthcare professional, someone on Healthy.me, someone from other online communities, or other); ii) closeness (Likert scale from 1 to 4); iii) frequency of discussing health matters with that person during that period (once or twice, three to four times, almost monthly, almost weekly, almost on a daily basis), and iv) helpfulness of advice sought/received (Likert scale from 1 to 4).

Additional measures were administered in the post-intervention questionnaire:

(4) *Help-seeking behaviors and health service utilization*: Help-seeking is defined as the behavior of actively seeking assistance [36], regardless of whether the source is informal or formal. A new scale was developed by the authors, adapted from the *Actual Help-seeking Questionnaire (AHSQ)* [36]. The scale covers help-seeking behaviors for physical and emotional well-being, informal and formal sources, as well as for self or others. While there are help-seeking scales relating to mental health help-seeking [36, 57], our review of the literature failed to locate a scale that measures help-seeking and health service utilization behaviors for both physical and emotional well-being. Our scale comprises nine items, three for each of the three areas: health service utilization, help-seeking for physical well-being, and help-seeking for emotional well-being. An example of a scale item for health

service utilization is “During the study, did you visit the University Counselling and Psychological Services?”, with dichotomous response options (Yes, No).

An example of a help-seeking scale item for physical / emotional well-being is “During the study, did you seek assistance in regards to physical [emotional] well-being concerns? (Note: help-seeking can range from talking to a friend to visiting a healthcare professional)”, with five response options: “Yes – for myself”, “Yes – for another person”, “Yes – for both myself and others”, “No – I did not seek assistance, but there was a physical well-being concern”, or “Not applicable – no physical well-being concerns”.

(5) *Feedback on Healthy.me*: participants were asked to provide feedback on their overall experience of using *Healthy.me*, as well as their feedback on specific features on the website, using a range of scale items such as Likert scale, free-text comments, and checkbox answer options.
