# Supplementary material for: Which Bundles of Features in a Web-Based Personally Controlled Health Management System Are Associated With Consumer Help-Seeking Behaviors for Physical and Emotional Well-Being?
Source: J Med Internet Res. 2013 May 6;15(5):e79. doi: 10.2196/jmir.2414 (PMC3650927; doi:10.2196/jmir.2414)
Supplement: Supplementary file 2 [file jmir_v15i5e79_app2.pdf]

Appendix 2. Details of logistic regression results for each outcome measure.

- *Visits to a healthcare professional*: after controlling for age and gender, the model explained between 12.5% (Cox and Snell R-square) and 16.8% (Nagelkerke R-square) of variance in whether participants visited a healthcare professional, and correctly classified 65.4% of cases (Appendix Table 1). Independent variables [ie, use of personal health record to add/update entries (OR = 2.82, 95% CI: 1.63 to 4.89), answered questions on poll (OR = 1.47, 95% CI: 1.02 to 2.12), gender (OR = 2.37, 95% CI: 1.71 to 3.30), and age (OR = 1.05, 95% CI: 1.03 to 1.07)] made a unique statistically significant contribution to the model (χ^2 (8) = 94.1, *P* <.001).
- *Visits to the University Health Service*: after controlling for age, gender and whether the participant was an existing patient at the University Health Service before the study, the model explained between 20.0% (Cox and Snell R-square) and 32.7% (Nagelkerke R-square) of variance in whether participants visited the University Health Service, and correctly classified 84.5% of cases (Appendix Table 2). Independent variables [ie use of online appointment booking service (OR = 1.74, 95% CI: 1.01 to 3.00), and existing patient at University Health Service (OR = 0.07, 95% CI: 0.04 to 0.11)] made a unique statistically significant contribution to the model (χ^2 (10) = 157.3, *P* <.001).
- *Visits to the University Counselling and Psychological Services*: after controlling for age, gender and whether the participant visited the University Counselling and Psychological Services prior to the study, the model explained between 7.5% (Cox and Snell R-square) and 21.5% (Nagelkerke R-square) of variance in whether participants visited the University Counselling and Psychological Services, and correctly classified 94.3% of cases (Appendix Table 3). Independent variables [ie, use of diary (OR = 4.92, 95% CI: 1.40 to 17.35), use of online appointment booking (OR = 6.04, 95% CI: 2.30 to 15.85), and prior visits to the

University Counselling and Psychological Services (OR = 0.11, 95% CI: 0.05 to 0.23)] made a unique statistically significant contribution to the model (χ^2 (9) = 54.7, *P* <.001).

- *Formal or informal help-seeking on physical well-being*: after controlling for age and gender, the model explained between 5.3% (Cox and Snell R-square) and 7.1% (Nagelkerke R-square) of variance in whether participants sought physical health assistance, and correctly classified 60.9% of cases (Appendix Table 4). Independent variables [ie accessing the personal health record (OR = 1.73, 95% CI: 1.18 to 2.53), age (OR = 1.02, 95% CI: 1.01 to 1.04), and gender (OR = 1.82, 95% CI: 1.33 to 2.49)] made a unique statistically significant contribution to the model (χ^2 (8) = 38.5, *P* <.001).
- *Formal or informal help-seeking on emotional well-being*: after controlling for age and gender, the model explained between 2.9% (Cox and Snell R-square) and 4.0% (Nagelkerke R-square) of variance in whether participants sought emotional health assistance, and correctly classified 68.4% of cases (Appendix Table 5). Independent variables [ie, answered poll questions (OR = 1.03, 95% CI: 1.00 to 1.05), and gender (OR = 1.67, 95% CI: 1.19 to 2.35)] made a unique statistically significant contribution to the model (χ^2 (8) = 20.5, *P* =.009).

Appendix Table 1. Logistic regression explaining PCHMS usage on likelihood of visiting a health care professional during study (after controlling for gender, age).

		B	S.E.	Wald	df	*P-value*	Adjusted Odds ratio	95.0% CI for Adjusted Odds Ratio	
								Lower	Upper
**Participant demographics**									
	Age	.050	.011	22.437	1	.000	1.052	1.030	1.074
	Gender [a]	.863	.168	26.365	1	.000	2.371	1.706	3.297
**PCHMS usage** [b]									
	Personal health record (PHR)	1.039	.280	13.816	1	.000	2.827	1.634	4.890
	Poll	.385	.186	4.301	1	.038	1.470	1.021	2.115
	Book appointment	-.572	.325	3.096	1	.079	.564	.298	1.067
	Journey	.175	.238	.543	1	.461	1.191	.748	1.898
	Diary	.541	.501	1.165	1	.280	1.717	.643	4.585
	Forum	.010	.372	.001	1	.978	1.010	.487	2.095

[a] Gender: 0=Female; 1=Male.
[b] PCHMS usage: 0=accessed; 1=did not access.

Appendix Table 2. Logistic regression explaining PCHMS usage on likelihood of visiting university health service during study (after controlling for participant demographics gender, age, existing patient at University Health Service before study).

		B	S.E.	Wald	df	*P-value*	Adjusted Odds ratio	95.0% CI for Adjusted Odds Ratio	
								Lower	Upper
**Participant demographics**									
	Age	-.006	.012	.240	1	.624	.994	.970	1.018
	Gender [a]	-.077	.240	.102	1	.749	.926	.579	1.482
	Existing patient at University Health Service [b]	-2.713	.239	128.920	1	.000	.066	.042	.106
**PCHMS usage** [c]									
	Personal health record (PHR)	.150	.081	3.436	1	.064	1.162	.991	1.362
	Poll	.000	.019	.000	1	.994	1.000	.964	1.038
	Book appointment	.551	.279	3.906	1	.048	1.735	1.005	2.995
	Journey	.066	.093	.492	1	.483	1.068	.889	1.282
	Diary	-.028	.207	.019	1	.891	.972	.648	1.458
	Forum	-.055	.175	.101	1	.751	.946	.672	1.332

[a] Gender: 0=Female; 1=Male.

[b] Existing patient at University Health Service: 0=No; 1=Yes.

[c] PCHMS usage: 0=accessed; 1=did not access.

Appendix Table 3. Logistic regression explaining PCHMS usage on likelihood of visiting the University Counselling and Psychological Services during study (after controlling for participant demographics gender, age, prior visits to the University Counselling and Psychological Services before study).

		B	S.E.	Wald	df	*P-value*	Adjusted Odds ratio	95.0% CI for Adjusted Odds Ratio	
								Lower	Upper
**Participant demographics**									
	Age	-.044	.029	2.209	1	.137	.957	.904	1.014
	Gender [a]	-.099	.384	.067	1	.796	.905	.427	1.921
	Prior visits to University Counselling and Psychological Services [b]	-2.198	.374	34.597	1	.000	.111	.053	.231
**PCHMS usage** [c]									
	Personal health record (PHR)	.177	.485	.133	1	.715	1.194	.461	3.088
	Poll	-.265	.428	.383	1	.536	.767	.332	1.775
	Book appointment	1.798	.492	13.346	1	.000	6.040	2.301	15.851
	Journey	-.754	.484	2.428	1	.119	.470	.182	1.215
	Diary	1.593	.643	6.137	1	.013	4.920	1.395	17.352
	Forum	-.602	.840	.513	1	.474	.548	.106	2.844

[a] Gender: 0=Female; 1=Male

[b] Prior visits to University Counselling and Psychological Services: 0=No; 1=Yes.

[c] PCHMS usage: 0=accessed; 1=did not access.

Appendix Table 4. Logistic regression explaining PCHMS usage on likelihood of seeking help (formal or informal) for physical well-being during study (after controlling for gender, age).

		B	S.E.	Wald	df	*P-value*	Adjusted Odds ratio	95.0% CI for Adjusted Odds Ratio	
								Lower	Upper
**Participant demographics**									
	Age	.024	.009	7.209	1	.007	1.024	1.006	1.042
	Gender [a]	.599	.160	14.048	1	.000	1.821	1.331	2.491
**PCHMS usage** [b]									
	Personal health record (PHR)	.548	.194	7.950	1	.005	1.729	1.182	2.530
	Poll	.165	.182	.814	1	.367	1.179	.825	1.685
	Book appointment	-.310	.311	.992	1	.319	.734	.399	1.350
	Journey	-.030	.235	.016	1	.900	.971	.613	1.538
	Diary	-.005	.303	.000	1	.987	.995	.549	1.802
	Forum	.025	.208	.015	1	.903	1.026	.682	1.542

[a] Gender: 0=Female; 1=Male.
[b] PCHMS usage: 0= accessed; 1=did not access.

Appendix Table 5. Logistic regression explaining PCHMS usage on likelihood of seeking help (formal or informal) for emotional well-being during study (after controlling for participant demographics gender, age).

		B	S.E.	Wald	df	*P-value*	Adjusted Odds ratio	95.0% CI for Adjusted Odds Ratio	
								Lower	Upper
**Participant demographics**									
	Age	-.008	.009	.660	1	.417	.992	.974	1.011
	Gender [a]	.514	.174	8.696	1	.003	1.672	1.188	2.354
**PCHMS usage** [b]									
	Personal health record (PHR)	-.010	.205	.002	1	.960	.990	.663	1.479
	Poll	.024	.012	4.285	1	.038	1.025	1.001	1.049
	Book appointment	.540	.310	3.033	1	.082	1.715	.935	3.149
	Journey	-.264	.243	1.182	1	.277	.768	.477	1.236
	Diary	-.186	.312	.353	1	.552	.830	.450	1.532
	Forum	.167	.216	.597	1	.440	1.182	.773	1.806

[a] Gender: 0=Female; 1=Male.
[b] PCHMS usage: 0=accessed; 1=did not access.
